# Comparative analysis of conventional penile clamps and Uriclak device in managing male incontinence following radical, turp, or laser prostate surgery

**DOI:** 10.3389/fsurg.2023.1301353

**Published:** 2023-12-19

**Authors:** Eva Ten

**Affiliations:** Department of Urology, Franziskus Hospital, Berlin, Germany

**Keywords:** prostate surgery, TURP, male urinary incontinence, cunningham clamp, Uriclak, penile clamp for incontinence, incontinence clamp, male incontinence device

## Abstract

This article examines the impact of various prostate surgery techniques on male urinary incontinence, evaluating the feasibility, efficacy, and safety of penile clamps as a post-prostate surgery treatment. The study compares the characteristics and applications of conventional penile clamps and the Uriclak urethral compression device, highlighting their differences and potential for managing male incontinence.

## Introduction

Whether dealing with prostate cancer or BPH, it's not uncommon for males to deal with urinary incontinence after prostate surgery. The prostate gland is vital for controlling urination, and surgical procedures can disrupt this intricate balance.

Prostate surgery often involves manipulating or removing part or all of the prostate gland, inadvertently affecting the urinary sphincter or the nerves and muscles controlling the bladder and urethra. This disruption can lead to varying degrees of urinary leakage or incontinence.

The severity and duration of urinary incontinence vary based on factors such as surgery type, overall health, pre-existing prostate conditions, and surgical approach. While incontinence might be temporary for some, it can persist for others, necessitating ongoing management and support.

Individuals who've undergone prostate surgery should collaborate with their healthcare team, including urologists and physiotherapists, to devise a tailored plan for managing urinary incontinence. This plan may involve exercises, lifestyle modifications, behavioral techniques, and, in some cases, medical devices or surgical interventions to enhance urinary control.

Understanding and addressing urinary incontinence post-prostate surgery are crucial aspects of post-operative care, aimed at improving the quality of life during recovery.

## Impact of prostate surgery on male incontinence

Despite advancements in prostate surgery techniques, is urinary incontinence still a common side effect? Analyzing male incontinence incidence in commonly used techniques, for both prostate cancer and BPH, provides insights.

Data from studies (1–3) are crucial.

**Open radical prostatectomy:** Eighty two percent immediately after surgery, with 60% experiencing stress incontinence in the following year.

**Modern radical prostatectomy techniques (including laparoscopic and/or robotic procedures):** Sixty percent experience stress incontinence immediately after surgery, with long-term rates at 5%–10%.

**TURP (only for BPH):** Between 30% and 40% experience incontinence immediately after surgery. However, late incontinence, persisting for more than 6 months, is only 0.5%.

**Holmium laser prostate enucleation (HoLEP) (only for BPH):** Recent reports indicate postoperative incontinence rates between 1.4% and 44%.

While HoLEP rates are higher than TURP, both techniques show negligible long-term urinary incontinence rates.

Study data also reveal that 30%–50% of patients experience urgency incontinence immediately after surgery. Continence improves substantially in all techniques one year after the intervention, except for laser procedures, where rates are not documented.

In summary, urinary incontinence remains a significant issue for men post-prostate surgery, especially in prostate cancer cases. Modern techniques with lower incontinence rates, like TURP and HoLEP, aren't applicable to prostate cancer. Study (2) suggests conservative treatment is the best option, proposing actions such as pelvic floor muscle training, electrostimulation, and the use of penile clamps. However, are penile clamps a safe and effective option compared to other devices? Do they improve the quality of life for stress and urgency incontinence patients?

## Understanding external clamps for incontinence and their advantages

Penile clamps, designed to compress the urethra, effectively control involuntary urine leakage. Their advantages (4–7) are noteworthy.

**Adaptability to varying incontinence levels:** Penile clamps are versatile, with different sizes and designs catering to individual patient needs. They address mild to severe incontinence, providing an adjustable level of control unmatched by other devices.

**Cost-effectiveness:** Compared to pricier surgical procedures and advanced treatments, clamps offer a cost-effective solution, appealing to those aiming to enhance their quality of life without excessive financial strain.

**Complication mitigation:** In contrast to urinary catheters, which heighten urinary tract infection risks with prolonged use, penile clamps, not requiring urethral insertion, significantly reduce this risk. Being a non-surgical alternative, they avoid the risks linked with more invasive incontinence treatments.

**Efficient incontinence control:** Purpose-built to control inadvertent urine loss in men, penile clamps offer a sense of normalcy and control not easily achieved with alternatives like adult diapers or catheters. By securely holding the penis, they prevent leaks, allowing patients to proceed with daily routines without concern.

## Side effects of penile clamps

Some studies (4, 5, 7) suggest the potential for penile inflammation and restricted blood circulation with excessive use. Following the manufacturers' instructions to reposition the clamps every 2/3 h (each time one urinates) helps prevent these effects (8).

## Uriclak vs. conventional devices

The conventional penile clamp features soft materials, typically in **rectangular, oval, or round shapes, with a lateral opening** for easy application and removal. It is also known as Cunningham clamps. Conversely, Uriclak, a flexible penile clamp resembling a flattened ring, lacks closures. By pressing it inward with one hand, it adopts an oval shape, facilitating placement or urination, and reverting to its flattened shape upon release.

## Results

The study (7) involved six men who underwent prostate surgery, evaluating four distinct commercial brands. Among these, three were Cunninghan clamps equipped with closures, while the remaining brand was the Uriclak urethral compression device. The study measured various variables, including circulatory impedance, inflammatory response, and urine leakage retention capacity.

According to the findings, all clamps demonstrated excellent tolerance, effectiveness, and safety during short usage periods. Intolerance and malfunctions (inadequate closure pressure) emerged as the primary reasons prompting patients to discontinue use. Uriclak emerged as the preferred choice due to its comfort and ease of use. However, it's worth noting that the study did not assess durations longer than 1 h, despite manufacturers recommending continuous usage with repositioning every 2 or 3 h (5). Throughout the study, incontinence clamps proved beneficial for stress and mixed incontinence. However, for cases of urge incontinence, the utility of clamps is constrained, as they do not eliminate the need to urinate, although they can assist patients in reaching the bathroom promptly.

## Conclusion

Even with modern surgical techniques, a substantial percentage of men undergoing prostatectomy experience urinary incontinence, which, though typically mild and temporary, significantly impacts their quality of life. Whether facing temporary or irreversible incontinence, penile clamps emerge as a viable treatment option for stress incontinence. Their effectiveness in control, coupled with comfort and versatility, establishes them as a preferred choice for many patients. Since each case of incontinence is different, personalized approaches may be needed, penile clamps stand as a practical, comfortable, and cost-effective option. Clamps for urinary incontinence prove to be both safe and effective, with side effects being virtually negligible when used within the recommended conditions and time limits stipulated by manufacturers. However, it's crucial to note that not all patients adapt to these devices, and discomfort due to excessive pressure often leads to discontinuation.

Uriclak, with its soft and flexible construction devoid of closures, distinguishes itself for its comfort and ease of use. Its innovative design presents a practical and comfortable splash-free solution for male incontinence, positioning it as a promising option in treatment.
